# Cathepsin B aggravates coxsackievirus B3-induced myocarditis through activating the inflammasome and promoting pyroptosis

**DOI:** 10.1371/journal.ppat.1006872

**Published:** 2018-01-23

**Authors:** Yaping Wang, Liangliang Jia, Jian Shen, Yidong Wang, Zurong Fu, Sheng-an Su, Zhejun Cai, Jian-an Wang, Meixiang Xiang

**Affiliations:** Department of Cardiology, Second Affiliated Hospital, Zhejiang University School of Medicine, Cardiovascular Key Lab of Zhejiang Province, Hangzhou, Zhejiang, China; University of California, Irvine, UNITED STATES

## Abstract

Cathepsin B (CatB) is a cysteine proteolytic enzyme widely expressed in various cells and mainly located in the lysosomes. It contributes to the pathogenesis and development of many diseases. However, the role of CatB in viral myocarditis (VMC) has never been elucidated. Here we generated the VMC model by intraperitoneal injection of coxsackievirus B3 (CVB3) into mice. At day 7 and day 28, we found CatB was significantly activated in hearts from VMC mice. Compared with the wild-type mice receiving equal amount of CVB3, genetic ablation of CatB (*Ctsb*^*-/-*^) significantly improved survival, reduced inflammatory cell infiltration, decreased serum level of cardiac troponin I, and ameliorated cardiac dysfunction, without altering virus titers in hearts. Conversely, genetic deletion of cystatin C (*Cstc*^*-/-*^), which markedly enhanced CatB levels in hearts, distinctly increased the severity of VMC. Furthermore, compared with the control, we found the inflammasome was activated in the hearts of wild-type mice with VMC, which was attenuated in the hearts of *Ctsb*^*-/-*^ mice but was further enhanced in *Cstc*^*-/-*^ mice. Consistently, the inflammasome-initiated pyroptosis was reduced in *Ctsb*^*-/-*^ mice hearts and further increased in *Cstc*^*-/-*^ mice. These results suggest that CatB aggravates CVB3-induced VMC probably through activating the inflammasome and promoting pyroptosis. This finding might provide a novel strategy for VMC treatment.

## Author summary

Severe VMC could lead to sudden cardiac death especially in youths, and is also the most common cause of secondary dilated cardiomyopathy. However, we still lack effective and specific clinical treatments currently. Therefore, further exploration of the pathogenesis and new therapeutic targets are urgently needed. Our results implied that CatB, a cysteine protease mainly located in the lysosome, is activated in the hearts of mice with VMC induced by intraperitoneal injection of CVB3. Genetic deletion of CatB significantly improves survival, attenuates cardiac inflammation, decreases serum cardiac troponin I levels and alleviates cardiac dysfunction, without altering virus titers in hearts. However, ablation of its main endogenous inhibitor, cystatin C, distinctly exaggerates the disease severity. Mechanistically, we found that CatB influences VMC probably by activating the NLRP3 inflammasome and promoting caspase-1-induced pyroptosis. This may provide a potential new therapeutic strategy for VMC.

## Introduction

Myocarditis, defined as a nonspecific inflammatory disease of the myocardium, is most commonly caused by cardiotropic virus infection, especially for coxsackieviruses[1, 2]. The clinical manifestations and severity vary among patients with viral myocarditis (VMC)[3]. Although some patients only present mild or even self-limited symptoms, VMC accounts for 8.6% to 12% of sudden cardiac deaths in young people due to its resultant acute heart failure or ventricular arrhythmias[4–6]. In addition, about 21% of patients with acute VMC may progress into dilated cardiomyopathy (DCM), which may lead to repeated heart failure and is the major reason for heart transplantation at present[7]. However, except for supportive care, no other effective and specific therapies are proven effective for clinical use currently[8].

Cathepsin B (CatB) is an intracellular cysteine protease, mainly localized in the lysosome[9]. By its involvement in many pathophysiologic processes including apoptosis, autophagy, extracellular matrix turnover, inflammation and immune responses, CatB plays an important role in many diseases, such as cancer, rheumatoid arthritis, cardiovascular diseases, etc[9–11]. It has also been demonstrated that CatB is involved in viral infectious diseases because of its relations with virus entry, replication as well as virus-mediated cell apoptosis and immune responses[12–14]. Specifically, a recent study showed that CatB was significantly upregulated in muscle tissues of both patients with polymyositis and Guinea pigs with Coxsackievirus B1-induced polymyositis, and administration of the CatB inhibitor attenuated inflammation and apoptosis in muscle tissues of Guinea pigs with polymyositis[14]. Considering the similarity between the pathophysiology of polymyositis and myocarditis, with an important inflammatory part in a context of viral infection in both cases, cumulated with the previous demonstration of the role of CatB in the former, we hypothesized that CatB might also participate in the pathogenesis of Coxsackievirus B3 (CVB3)-induced myocarditis.

The inflammasome is an intracellular multiprotein complex consisting of three components: a cytosolic pattern recognition receptor, the adaptor protein ASC (apoptosis-related speck-like protein containing a caspase recruitment domain) and the cysteine protease procaspase-1[15]. The nucleotide-binding oligomerization domain (NOD)-like receptor family, pyrin domain-containing protein 3 (NLRP3) is the most studied pattern recognition receptor[15]. Upon activation, NLRP3 recruits ASC, which further recruits procaspase-1. Activation of procaspase-1 can cleave pro-interleukin (IL)-1β and pro-IL-18 into mature IL-1β and IL-18, which are then released into circulation to amply the inflammatory responses. In addition, activated caspase-1 can also initiate a specific form of programmed cell death called pyroptosis[15]. Different from apoptosis, pyroptosis is a death pathway accompanied by release of a number of inflammatory cytokines, mainly including IL-1β and IL-18[16]. Specifically, the activated capase-1 cleaves gasdermin D, releasing its N-terminal domain, which oligomerizes in the membranes to form large pores causing subsequent membrane rupture and cell death[17]. The inflammasome has been implicated in many inflammation-related diseases, such as myocardial infarction and ischemia-reperfusion injury[18, 19]. Recently, formation of the inflammasome has also been found in VMC both in patients and mice[20, 21]. Besides, blockade of inflammasome activation by treating CVB3-inoculated mice with caspase-1 inhibitor Ac-YVAD-CHO significantly attenuated the severity of VMC[21].

According to previous data, CatB released from the lysosome is considered one of the upstream activators of the NLRP3 inflammasome[22]. Extracellular stimuli, including viral infection, could damage the lysosomes and release the lysosomal contents, including CatB, into the cytosol[23]. These findings suggest that CatB may exaggerate VMC via regulating the activation of the inflammasome.

In this study, we built the murine VMC model by intraperitoneal injection of CVB3, and investigated whether and how CatB contributed to VMC development, using genetically CatB knockout (*Ctsb*^*-/-*^) mice, as well as the mice deficient in cystatin C, which is an endogenous inhibitor of papain-like cysteine cathespins, particularly potent for CatB[24].

## Results

### CatB is activated in both acute and chronic phases of CVB3-induced VMC

**Fig 1:**
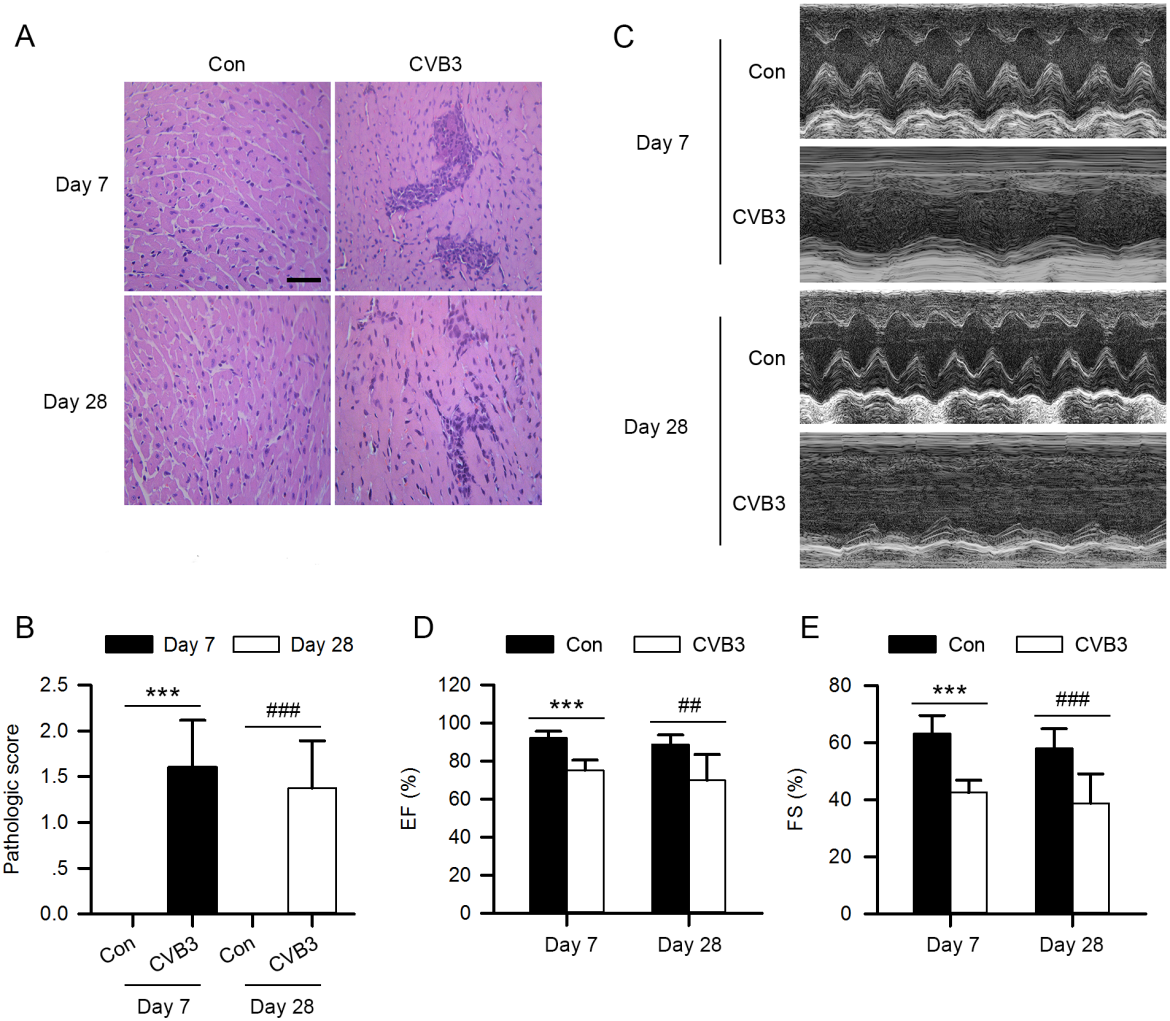
The establishment of CVB3-induced VMC model. 4-week-old male C57BL/6 mice were injected intraperitoneally with 1000 TCID_50_ of CVB3 virus or an equal amount of DMEM on day 0. (A) Representative HE staining of paraffin sections of heart tissues from VMC mice and control mice on both day 7 and day 28 after virus injection. Scale bar: 50μm. (B) The pathologic score of myocarditis was assessed according to the mononuclear infiltration foci and myocardial necrosis. (C) Representative M-mode echocardiographic images of VMC mice and control mice on day 7 and day 28 post-infection. (D, E) Left ventricular ejection fraction (LVEF) and left ventricular fractional shortening (LVFS) from the echocardiographic data for both day 7 and day 28 post-infection. (Con: control group; CVB3: coxsackievirus B3 group; Con: n = 10; CVB3: n = 10; ***P<0.001 *vs*. Con-day 7; ##P<0.01 *vs*. Con-day 28; ###P<0.001 *vs*. Con-day 28).

To investigate the role of CatB in VMC, we first generated the VMC model by intraperitoneal injection of 1000 TCID_50_ of CVB3 into 4-week-old male C57BL/6 mice. Transthoracic echocardiography was conducted, and mice were sacrificed on day 7 and day 28 postinfection (pi). At both time points, the hematoxylin and eosin (HE) staining showed apparent inflammatory infiltrates, and the echocardiography exhibited impaired cardiac function as evidenced by decreased ejection fraction (EF) and fractional shortening (FS) in the model group compared with the control (Fig 1, S1 and S2 Tables). These results suggest the successful establishment of CVB3-induced myocarditis.

**Fig 2:**
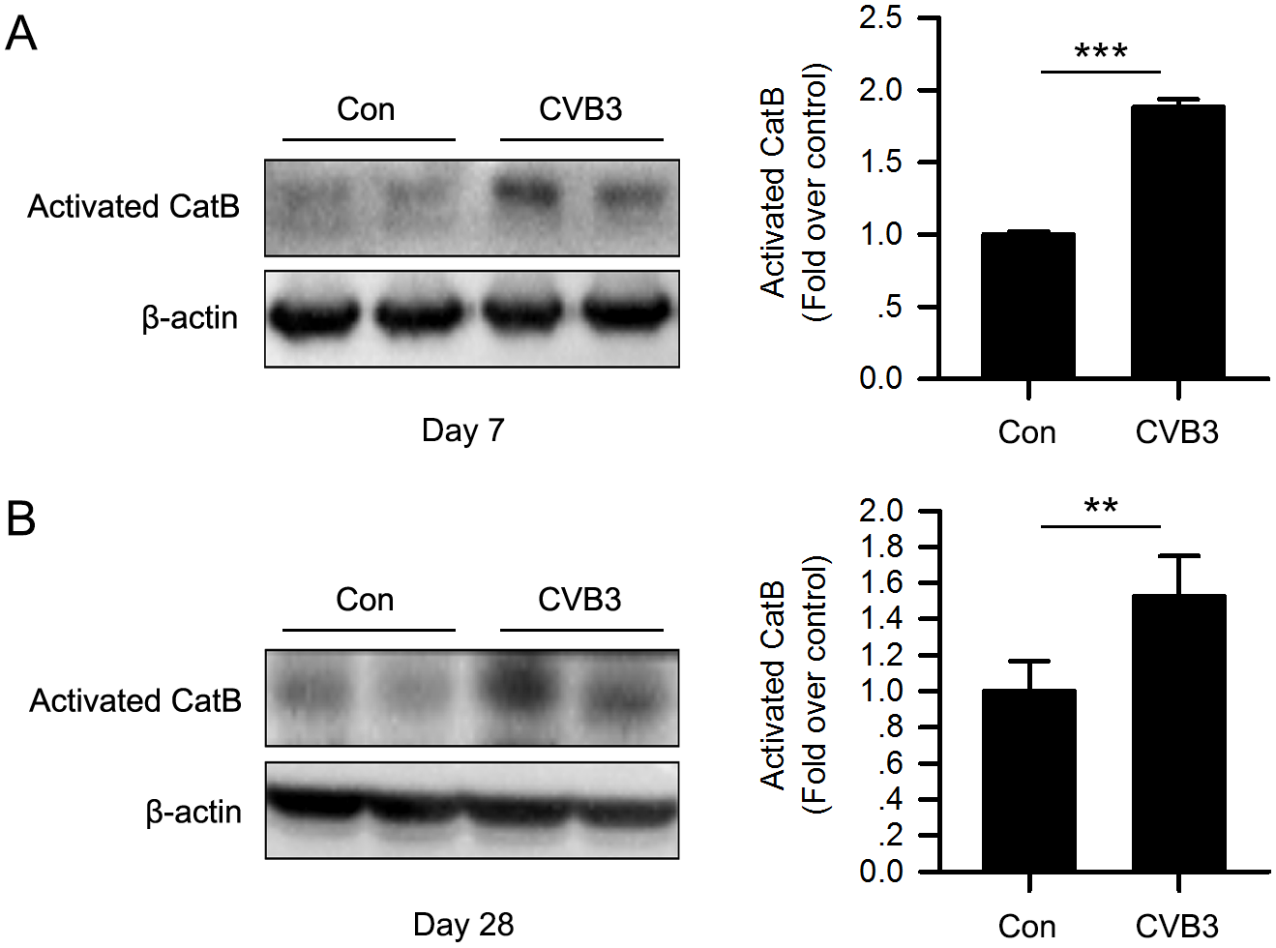
CatB was activated in hearts of CVB3-induced VMC mice. Hearts were harvested on day 7 and day 28 after virus inoculation. Western blot analysis showed that cardiac expression of activated CatB was significantly enhanced in the CVB3 group on day 7 (A) and day 28 (B) post-infection compared with the control group. (Con: control group; CVB3: coxsackievirus B3 group; Con: n = 10; CVB3: n = 10; **P<0.01 *vs*. Con; ***P<0.001 *vs*. Con).

Next, we detected the expression and activity of CatB in the mice hearts. As shown in Fig 2, the expression of activated CatB was significantly increased in CVB3-infected mice on both day 7 and day 28 pi. Besides, cardiac CatB activity was also significantly enhanced 7 days after virus inoculation (S1B Fig). This implies that CatB is probably involved in the pathogenesis of VMC.

### CatB exacerbates CVB3-induced cardiomyocyte damage and cardiac dysfunction

**Fig 3:**
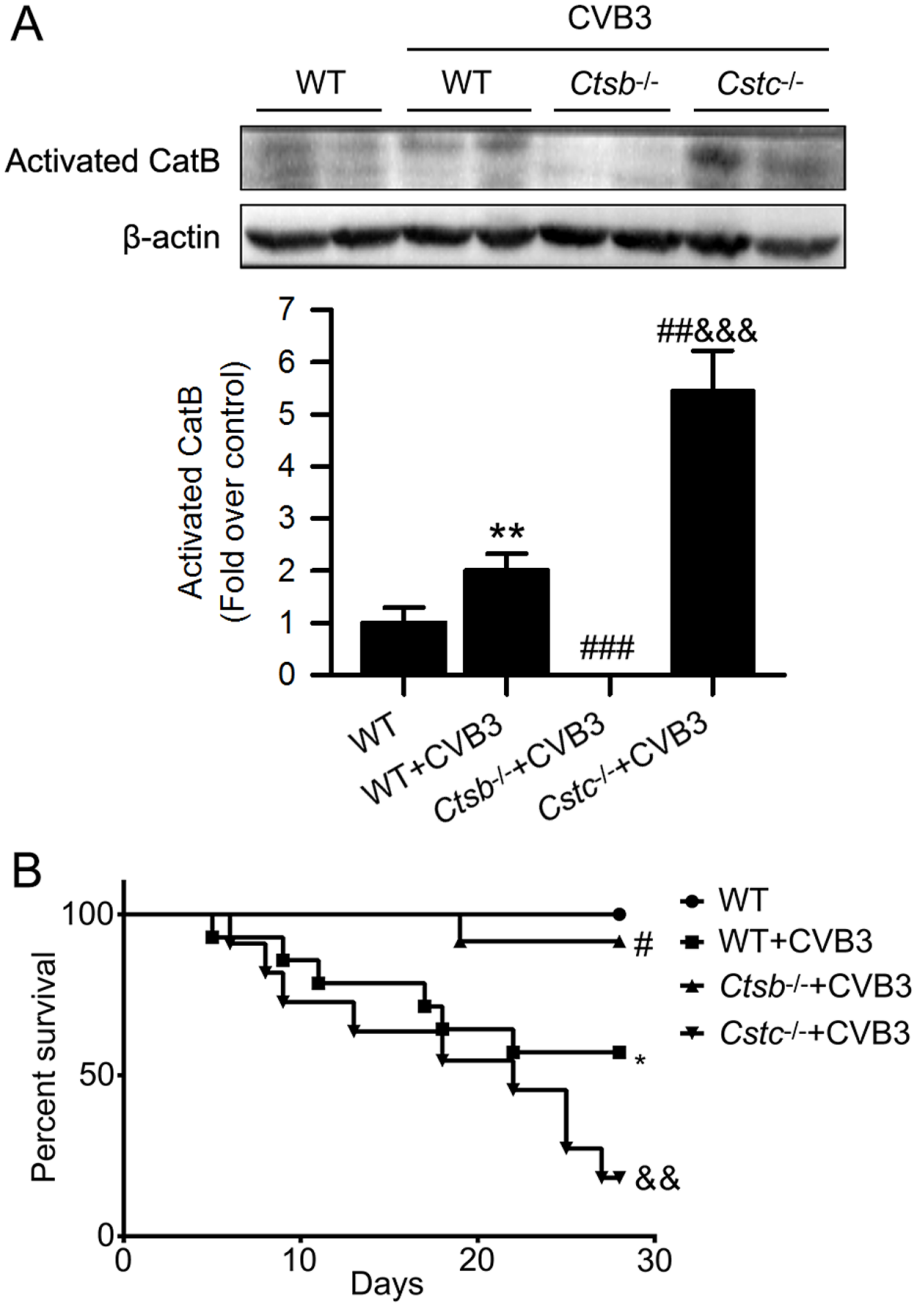
CatB deficiency increased while cystatin C deficiency decreased the survival of CVB3-induced VMC. Mice were randomly assigned into 4 groups: WT group (n = 10); WT+CVB3 group (n = 14); *Ctsb*^*-/-*^+CVB3 group (n = 12) and *Cstc*^*-/-*^+CVB3 group (n = 11). Each mouse of the latter three groups received 1000 TCID_50_ of CVB3 intraperitoneally, while the WT group received an equal amount of DMEM. (A) Western blot analysis of actived CatB expression to verify the deletion and overexpression of CatB in *Ctsb*^*-/-*^ mice and *Cstc*^*-/-*^ mice. (B) Survival was monitored daily until day 28 post-infection. The Kaplan-Meier curve showed a significant increase in survival in *Ctsb*^*-/-*^+CVB3 group and a remarkable decrease in *Cstc*^*-/-*^+CVB3 group, compared with WT+CVB3 group. (WT: wild-type; *P<0.05 *vs*. WT group; #P<0.05 *vs*. WT+CVB3 group; &&P<0.01 *vs*. *Ctsb*^*-/-*^+CVB3 group).

**Fig 4:**
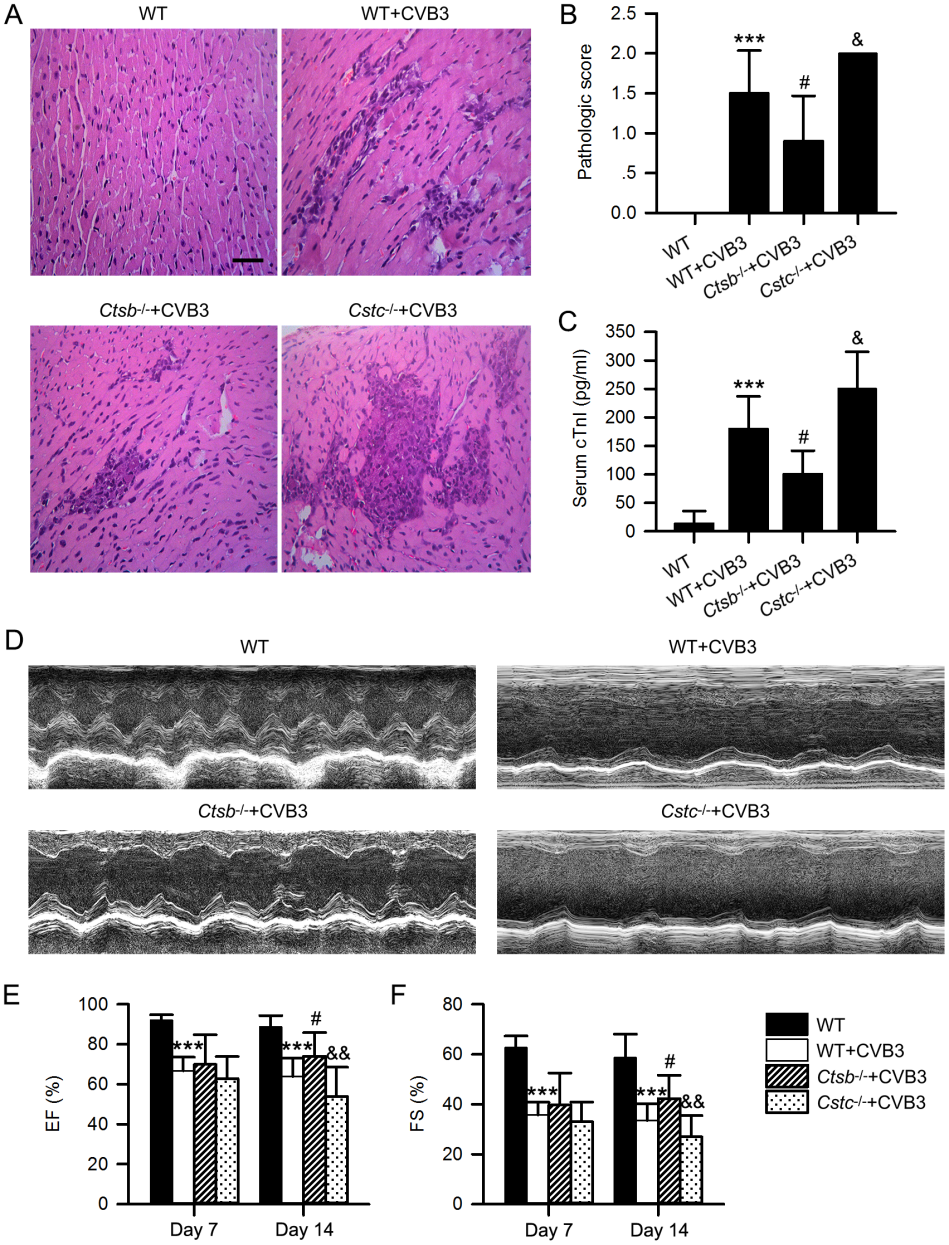
CatB deficiency alleviated while cystatin C deficiency aggravated the severity of CVB3-induced VMC. (A, B) Representative HE staining in hearts from mice of indicated intervention. Scale bar: 50μm. The HE staining showed the infiltration of inflammatory cells and pathologic score was significantly decreased in *Ctsb*^*-/-*^+CVB3 group while increased in *Cstc*^*-/-*^+CVB3 group on day 7 pi, compared with WT+CVB3 group. (C) The serum cTnI level was significantly lower in *Ctsb*^*-/-*^+CVB3 group and higher in *Cstc*^*-/-*^+CVB3 group than the WT+CVB3 group on day 7 pi. (D-F) The echocardiographic data showed that cardiac dysfunction caused by CVB3 infection was significantly alleviated in *Ctsb*^*-/-*^+CVB3 group, though there was no statistical difference between the *Cstc*^*-/-*^+CVB3 group and the WT+CVB3 group. (WT group: n = 10; WT+CVB3 group: n = 14; *Ctsb*^*-/-*^+CVB3 group: n = 12; *Cstc*^*-/-*^+CVB3 group: n = 11; ***P<0.001 *vs*. WT group; #P<0.05 *vs*. WT+CVB3 group; &P<0.05 *vs*. *Ctsb*^*-/-*^+CVB3 group; &&P<0.01 *vs*. *Ctsb*^*-/-*^+CVB3 group).

To further explore the role of CatB in VMC, we used *Ctsb*^*-/-*^ mice lacking CatB and cystatin C deficient (*Cstc*^*-/-*^) mice which overexpress cathepsins to directly investigate the impact of CatB on VMC. The deletion and overexpression of CatB in *Ctsb*^*-/-*^ and *Cstc*^*-/-*^ mice were verified by western blot (Fig 3A) and CatB enzymatic activity assay (S1 Fig). Then, we compared cardiac structure, cardiac function and survival among the *Ctsb*^*-/-*^, *Cstc*^*-/-*^ and wildtype (WT) mice, and found no significant differences in their baseline conditions (S2 Fig). Compared with 57.14% in the WT+CVB3 group, the survival rate up to 28 days was significantly increased to 91.67% in the *Ctsb*^*-/-*^+CVB3 group but dramatically decreased to 18.18% in the *Cstc*^*-/-*^+CVB3 group (Fig 3B). Moreover, *Ctsb*^*-/-*^ mice showed less inflammatory cell infiltration and lower pathologic scores whereas the *Cstc*^*-/-*^ mice exhibited the contrary results on day 7 pi, compared with the WT+CVB3 group (Fig 4A and 4B).

Serum cardiac troponin I (cTnI) level is a sensitive indicator of myocardial injury. As expected, on day 7 pi, the serum cTnI level was lower in *Ctsb*^*-/-*^ group but higher in *Cstc*^*-/-*^ group than that in WT+CVB3 group (Fig 4C).

We further assayed the effect of CatB on CVB3-mediated cardiac dysfunction. Deficiency of CatB significantly improved EF and FS compared with the WT+CVB3 group. However, accompanied with overexpressed CatB, *Cstc*^*-/-*^ mice had reduced cardiac contractility compared with the WT+CVB3 group (Fig 4D–4F, S3 Table). Together, these data indicate that CatB promotes VMC.

### Involvement of CatB in CVB3-induced VMC is independent of altering viral replication

It has been reported that CatB promotes entry and replication of several viruses[12, 25]. Here we tested if CatB affects CVB3 titers in hearts. As depicted in S3 Fig, the cardiac virus titers exhibited no significant difference among the WT+CVB3, *Ctsb*^*-/-*^+CVB3 and *Cstc*^*-/-*^+CVB3 groups on both day 7 and day 28 pi. These data imply that CatB promotes CVB3-induced VMC independently of altering viral replication.

### CatB enhances the activation of the NLRP3 inflammasome in CVB3-induced myocarditis

**Fig 5:**
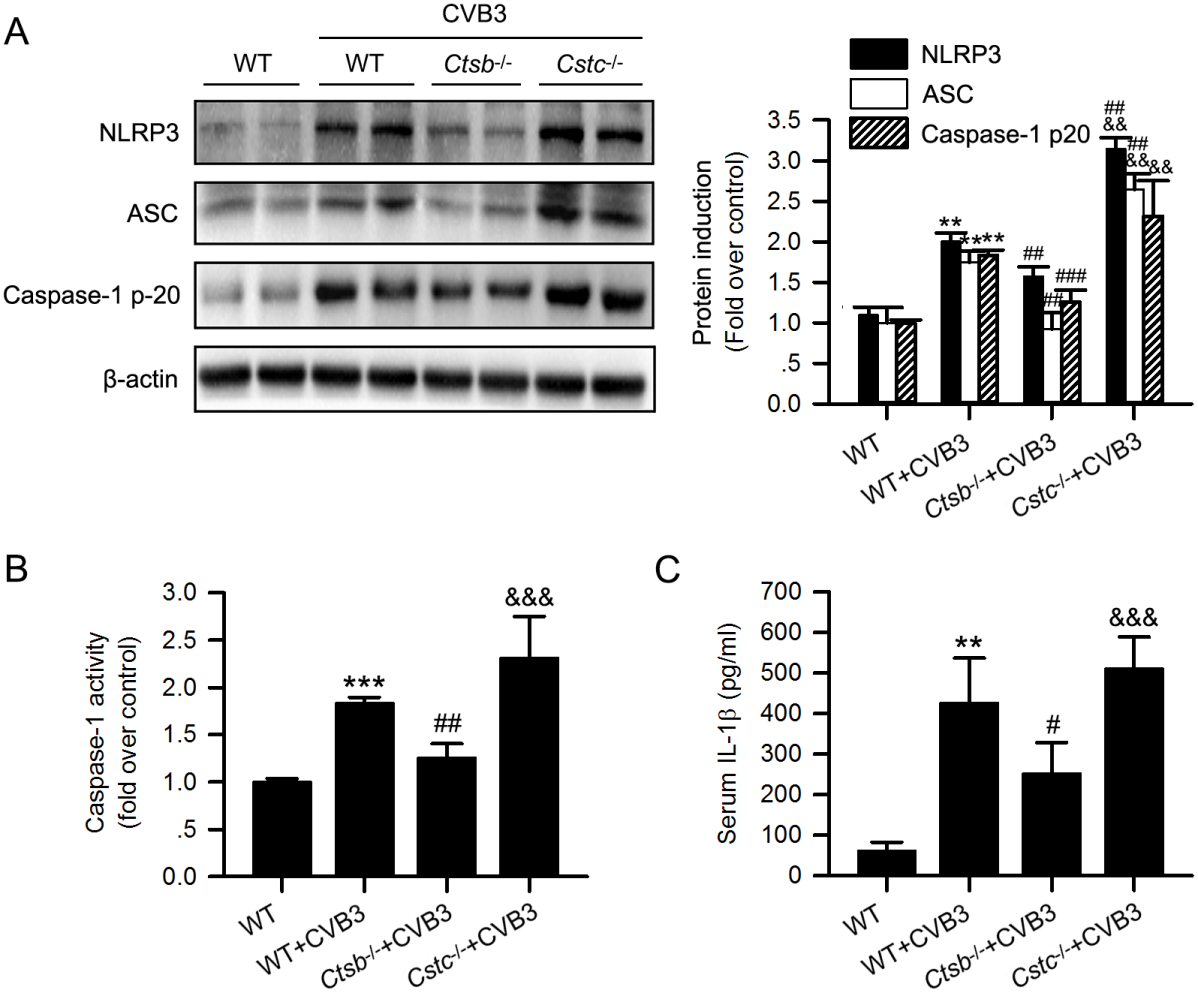
CatB deficiency reduced while cystatin C deficiency increased the activation of the NLRP3 inflammasome induced by CVB3 infection. (A) Western blot analysis of the expression of the components of the inflammasome including NLRP3, ASC, caspase-1 p-20 in hearts from mice receiving different treatments. (B) Cardiac caspase-1 activity was determined by a commercial assay kit and presented as fold change compared with the WT mice receiving DMEM. (C) Compared with WT+CVB3 group, CatB deficiency significantly decreased the serum IL-1β level while cystatin C deletion increased the level of IL-1β. (WT group: n = 10; WT+CVB3 group: n = 14; *Ctsb*^*-/-*^+CVB3 group: n = 12; *Cstc*^*-/-*^+CVB3 group: n = 11; **P<0.01 *vs*. WT group; ***P<0.001 *vs*. WT group; #P<0.05 *vs*. WT+CVB3 group; ##P<0.01 *vs*. WT+CVB3 group; ###P<0.001 *vs*. WT+CVB3 group; &&P<0.01 *vs*. *Ctsb*^*-/-*^+CVB3 group; &&&P<0.001 *vs*. *Ctsb*^*-/-*^+CVB3 group).

Recent studies have demonstrated that the NLRP3 inflammasome was activated in the myocardium of both patients and mice with VMC, and inhibiting caspase-1 activity significantly alleviated VMC[20, 21]. As CatB releasing from damaged lysosomes could activate the NLRP3 inflammasome[26], we hypothesized CatB might aggravate VMC through the NLRP3 inflammasome activation. The protein levels of NLRP3, ASC, caspase-1 p20, and serum IL-1β levels had no difference at baseline of the uninfected knockout mice and WT mice (S4A and S4C Fig). Because only two mice of the *Cstc*^*-/-*^ group survived till day 28 pi, we examined the levels of the components of the inflammasome in the mice hearts harvested on day 7. The levels of these components of the NLRP3 inflammasome were markedly increased in the WT+CVB3 group compared with the WT group, suggesting the activation of the NLRP3 inflammasome in VMC. Moreover, compared with the WT+CVB3 group, their levels were significantly decreased in the *Ctsb*^*-/-*^ group, but apparently enhanced in the *Cstc*^*-/-*^ group (Fig 5A and 5C).

### CatB promotes myocardial pyroptosis in CVB3-induced myocarditis

**Fig 6:**
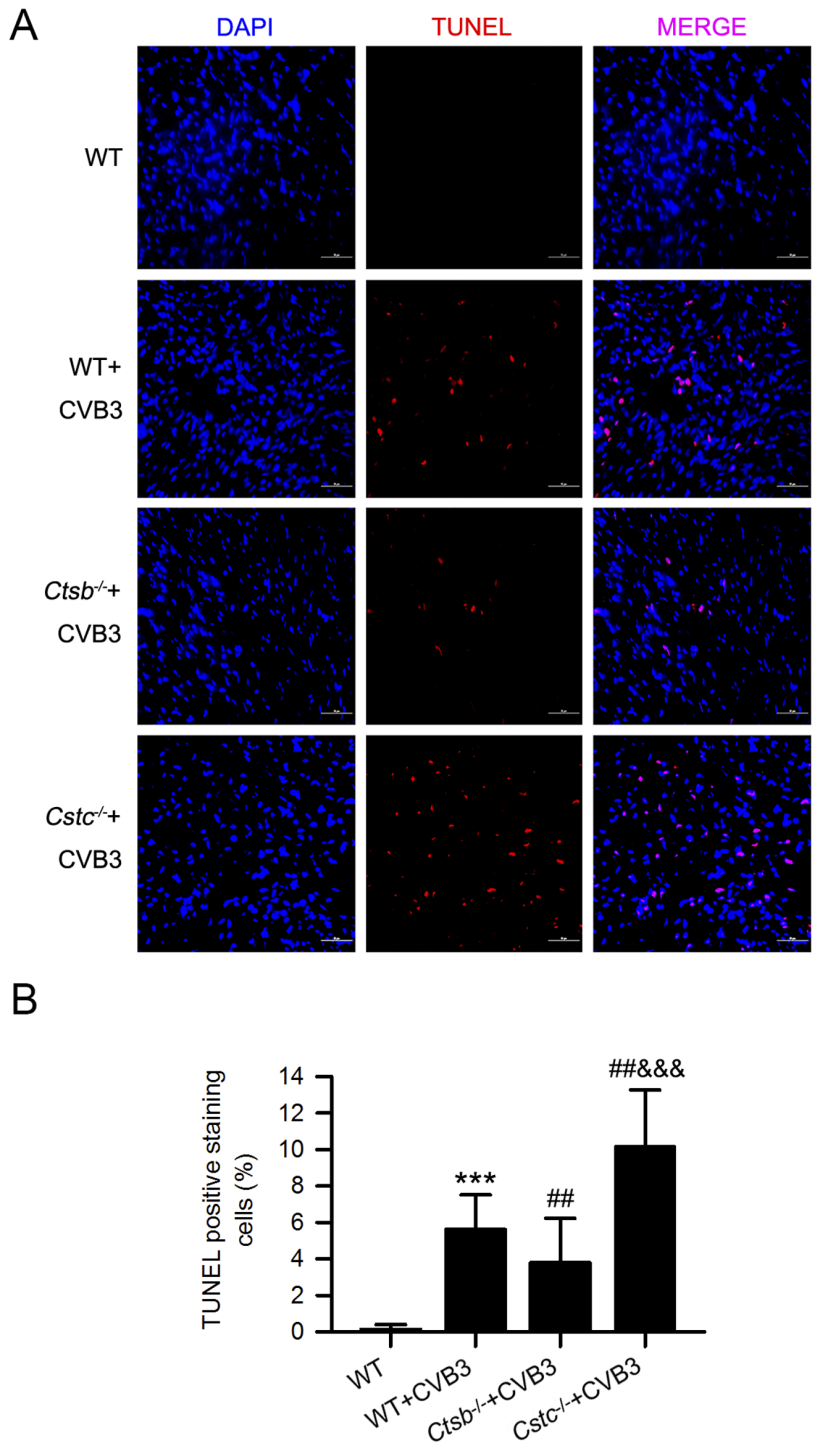
CatB deficiency reduced while cystatin C deficiency increased myocardial pyroptosis caused by CVB3 inoculation. (A) Representative cardiac TUNEL staining of mice with different treatments. Scale bar: 50μm. (B) The statistical result showed the increased TUNEL positive staining cells in the WT+CVB3 group were significantly decreased in the *Ctsb*^*-/-*^+CVB3 group, but further increased in the *Cstc*^*-/-*^+CVB3 group. (WT group: n = 10; WT+CVB3 group: n = 14; *Ctsb*^*-/-*^+CVB3 group: n = 12; *Cstc*^*-/-*^+CVB3 group: n = 11; ***P<0.001 *vs*. WT group; ##P<0.01 *vs*. WT+CVB3 group; &&&P<0.001 *vs*. *Ctsb*^*-/-*^+CVB3 group).

Pyroptosis is a newly discovered form of programmed cell death dependent on the activation of the inflammatory caspases, including caspase-1[27]. We found the enhanced caspase-1 activity in WT+CVB3 group was significantly decreased in the *Ctsb*^*-/-*^ group but further increased in the *Cstc*^*-/-*^ group (Fig 5B). In addition, the caspase-1-induced pyroptosis was detected by the TUNEL staining, which showed less cell death in the *Ctsb*^*-/-*^+CVB3 group but more in the *Cstc*^*-/-*^+CVB3 group, compared with the WT+CVB3 group (Fig 6). The levels of caspase-1 activity and cell death detected by TUNEL showed no difference among the uninfected WT, *Ctsb*^*-/-*^ and *Cstc*^*-/-*^ mice (S4B and S5 Figs).

## Discussion

The present study determined the role of CatB in CVB3-induced myocarditis. We found that CatB was activated in the hearts of CVB3-infected mice both in the acute and chronic phases, accompanied by the activation of the inflammasome. CatB deficiency markedly suppressed the activation of the inflammasome, reduced caspase-1-induced pyroptosis, attenuated cardiac inflammation, alleviated cardiomyocyte injury, prevented cardiac dysfunction and improved survival. In contrast, ablation of cystatin C significantly increased the expression of CatB, promoted the activation of the inflammasome, enhanced myocardial pyroptosis, and increased the severity of VMC. Based on these results, we concluded that CatB aggravated CVB3-induced myocarditis probably by activating the inflammasome and promoting pyroptosis.

The lysosome is a ubiquitous intracellular organelle essential for cell homeostasis. It participates in degradation of macromolecules, endocytosis, autophagy, lysosomal exocytosis, and cell death signaling[28]. These functions of the lysosome largely depend on the hydrolases it contains[28]. CatB is one of the most important lysosomal cysteine proteases with highest activity in the acidic environment[29]. Besides the environmental pH value, CatB activity is mainly influenced by its inhibitors. Cystatin C is the most important endogenous inhibitor of CatB, both inhibiting its activity and synthesis[30]. Extensive studies have documented the crucial involvement of CatB in many diseases, including viral infection[31–33]. For example, Kartik Chandran et al. found that proteolysis of virus glycoprotein 1 by CatB was necessary for the entry of Ebola virus into the host cell[12]. In another study, expression of CatB was found dramatically increased in Dengue virus (DENV)-infected HepG2 cells, and both treating with CatB inhibitor and RNAi knockdown of CatB reduced the level of cleaved caspase-3, suggesting a role of CatB in DENV-induced apoptosis[34]. Moreover, CatB level was also increased in both muscle and lung tissues of Guinea pigs with CVB1-induced polymyositis, and inhibition of CatB with CA-074Me exerted a protective effect by alleviating inflammation and apoptosis[14, 35]. Our research demonstrates that myocardial activated CatB levels are enhanced both in the acute and chronic phases of CVB3-induced VMC. Absence of CatB significantly attenuates, while overexpression of CatB by ablation of cystatin C exacerbates CVB3-induced VMC.

According to previous data, the possible mechanisms underlying CatB-mediated effects include: degradation of extracellular matrix (ECM), induction of cell death, activation of the inflammasome, participation in autophagy etc[22, 34, 36–39]. Many pathogens, including viruses, can activate the inflammasome and induce pyroptosis[40]. Our study focused on the effect of CatB on the inflammasome activation during CVB3 infection. Consistent with a previous study[21], our data showed that the inflammasome was activated and the inflammasome-induced pyroptosis was increased in the hearts of CVB3-inoculated mice. Furthermore, this phenomenon was blocked in *Ctsb*^*-/-*^ mice but more apparent in *Cstc*^*-/-*^ mice, suggesting that CatB promotes the activation of the inflammasome and pyroptosis in VMC. It has been verified that inhibiting inflammasome activation by treating mice with caspase-1 inhibitor significantly alleviated CVB3-induced myocarditis[21]. A combination of this fact and our results confirmed our hypothesis that CatB exaggerated VMC partially by regulating the activation of the inflammasome and its resultant pyroptosis.

Cardiac viral load is one of the key factors that determine the severity and progress of VMC. The effect of CatB on virus replication is rather complicated. One study proved that, compared with the WT mice, *Ctsb*^*-/-*^ mice showed no difference in virus replication and time to death when challenged with lethal mouse-adapted Zaire ebolavirus[41]. In our CVB3-induced VMC model, we demonstrate that deletion of CatB and cystatin C had no impact on cardiac virus titers of mice with VMC on both day 7 and day 28 pi. This suggests that the influence of CatB on VMC was independent of affecting CVB3 replication.

Our results reveal the pathogenic role of CatB in CVB3-induced myocarditis, suggesting that inhibition of CatB could represent a promising treatment for VMC. In fact, CatB has been a hopeful target for pharmacological therapy of several kinds of diseases[42, 43]. Treatment with the CatB selective inhibitor CA074 greatly suppressed bone metastasis of breast cancer in a 4T1.2 murine model[43]. The broad-spectrum cathepsin inhibitor E64d and the specific CatB inhibitor CA-074Me were both proved capable of reducing brain β-amyloid peptides and improving memory in the murine Alzheimer’s disease model[42]. The therapeutic effect of these inhibitors on VMC could also be investigated, and this may provide a new approach to treating VMC.

However, there are still a few limitations of our study. First, cystatin C is the endogenous inhibitor of papain-like cysteine cathepsins, but not specific for CatB. Thus, the aggravation of the disease severity in infected cystatin C knockout mice is a comprehensive result of overexpression of several kinds of cathepsins, but not solely due to the increased levels of CatB. Second, deficiency of CatB and cystatin C had no effect on cardiac CVB3 replication 7 and 28 days after virus infection, and we only determined the cardiac function within 28 days pi in our study. It is still necessary to detect cardiac virus titers and cardiac function in a longer time. Third, as far as we know, in addition to caspase-1 activation, there is no any other specific strategy to detect pyroptosis. We used TUNEL staining, besides caspase-1 activation, to demonstrate pyroptosis which is in accordance with other reports[44, 45], but the specificity may be limited.

To sum up, our study demonstrates that CatB aggravates CVB3-induced myocarditis and the one pathway, induction of the inflammasome and pyroptosis, has been shown to be a result of cathepsin B activity in this murine model of VMC. CatB may be a potential therapeutic target against VMC.

## Materials and methods

### Ethics statement

All animal experiments were approved by the ethical board of the Animal Care and Use Committee of Zhejiang University (zju201308-1-01-085), and were performed according to Guide for the Care and Use of Laboratory Animals of the U.S. National Institutes of Health. All efforts were made to minimize the number of animals and their suffering.

### Mice

The male C57BL/6 mice were purchased from Shanghai Slac Laboratory Animal Co. Ltd (Shanghai, China). The breeding pairs of both *Ctsb*^*-/-*^ mice and *Cstc*^*-/-*^ mice in C57BL/6J background were provided by Professor Guo-ping Shi (Harvard Medical School, MA, USA). The *Ctsb*^*-/-*^ mice were generated in the laboratory of Professor Shi[46], and the *Cstc*^*-/-*^ mice were bought from the Jackson Laboratories[47]. Mice at 4 weeks of age were used in all experiments.

### HeLa cells and virus

HeLa cells were purchased from American Type Culture Collection (ATCC) and cultured in Dulbecco’s modified eagle medium (DMEM) with high glucose (Shanghai Pufei Biotech Co., Ltd, China). The CVB3 (3m strain, a mutant of Nancy strain) was purchased from Wuhan Institute of Virology, Chinese Academy of Sciences, and preserved in Institute of Hypertension and Department of Internal Medicine, Tongji Hospital, Tongji medical college, Huazhong University of Science and Technology, China and stored at -80°C. The virus was amplified by infecting HeLa cells, subsequent freeze-thaw cycles and collection of the supernatants containing viruses. The virus titer was determined by a 50% tissue culture infectious dose (TCID_50_) assay of HeLa cell monolayer as previously described[48].

### Animal grouping and virus infection

To investigate the involvement of CatB in VMC, we randomly assigned the 4-week-old male C57BL/6 mice into two groups: control group (n = 10) and CVB3 group (n = 10). Then, to further evaluate the effect of CatB on the severity of VMC, mice were divided into four groups: WT group (n = 10, 4-week-old male C57BL/6 mice), WT+CVB3 group (n = 14, 4-week-old male C57BL/6 mice), *Ctsb*^*-/-*^+CVB3 group (n = 12, 4-week-old male *Ctsb*^*-/-*^ mice), *Cstc*^*-/-*^+CVB3 group (n = 11, 4-week-old male *Cstc*^*-/-*^ mice). Each mouse of the CVB3 groups was intraperitoneally injected with 1000 TCID_50_ of CVB3 to induce VMC, while the other groups received an equal amount of DMEM.

### Echocardiography

Transthoracic echocardiography was performed using the Vevo 2100 ultrasound imaging system (VisualSonics, Toronto, Canada) after anesthetization by isoflurane inhalation on day 7, day 14 and day 28 following virus injection. The echocardiographic data such as left ventricular ejection fraction (LVEF) and left ventricular fractional shortening (LVFS) were then measured blindly according to the operator’s manual.

### Histology and myocarditis grading

Mice were sacrificed and their hearts were harvested on day 7 and day 28 after CVB3 infection. The hearts were fixed in 10% phosphate-buffered formalin, embedded in paraffin, sectioned and then stained with hematoxylin and eosin. As previously described, the severity of myocarditis was assessed using a 0–4 scale, in which 0 = no inflammation; 1 = one to five distinct mononuclear inflammatory foci with involvement of 5% or less of the cross-sectional area; 2 = more than five distinct mononuclear inflammatory foci, or involvement of between 5% and 20% of the cross-sectional area; 3 = diffuse mononuclear inflammation involving over 20% of the area, without necrosis; 4 = diffuse inflammation with necrosis[49].

### Quantification of virus titers in mice hearts

Mouse heart tissues were aseptically obtained, weighed and homogenized in DMEM. After repeated freeze-thaw cycles, the samples were centrifuged at 300xg for 10 minutes, and the supernatants were collected. Then, the supernatants were used to determine the virus titers as previously described[48].

### Determination of serum cTnI and IL-1β levels

Levels of serum cTnI and IL-1β were determined using the commercial enzyme-linked immunosorbent assay (ELISA) kits (Cloud-Clone Corporation, Houston, TX) respectively, following the manufacturer’s instruction manuals.

### Western blot analysis

The frozen heart tissues were homogenized and then lysed using RIPA lysis buffer (Beyotime Biotechnology, China) added with the protease inhibitor (Thermo Fisher Scientific, MA). Protein samples were separated by SDS-PAGE electrophoresis and transferred to polyvinylidene difluoride (PVDF) membranes. The membranes were incubated with primary antibodies overnight and then with secondary antibodies for another hour. Finally, the bands were visualized using ECL solution (Merk Millipore, MA). The following antibodies were used: CatB antibody (1:200 dilution) from Santa Cruz, NLRP3 antibody (1:1000 dilution) from Abcam, ASC antibody (1:1000 dilution) from Millipore, caspase-1 antibody (1:1000 dilution) from Abcam, β-actin antibody (1:3000 dilution) from Santa Cruz, Horseradish peroxidase (HRP)- conjugated anti-rabbit and anti-mouse IgG (1:3000) from Santa Cruz. Densitometric quantification of the bands were performed using ImagePro Plus (Media Cybernetics, Warrendale, PA).

### Caspase-1 activity assay

The commercial Caspase-1 Activity Assay Kit (Beyotime Biotechnology, China) was applied to detect the caspase-1 activity in heart tissues. The tissue lysates were centrifuged at 16,000–20,000 g for 15 minutes, and the supernatants were collected. Meanwhile, an appropriate amount of the supernatants from each sample were incubated with the substrate Ac-YVAD-pNA in a 96-well plate for 60–120 minutes at 37°C. The absorbance values of the products pNA at 405 nm (OD_405_) were measured by using the microplate reader (Bio-Rad, Hercules, CA). Then, the activities of caspase-1 were calculated based on the above results and were finally shown as fold changes compared with control.

### CatB activity assay

Cardiac CatB activity was determined by using a commercial Cathepsin B Activity Assay Kit (Fluorometric) (Abcam, England) according to the instructions. The appropriate amount of heart tissues lysates was incubated with CatB substrate in the 96-well black plates with clear bottoms for 60–120 minutes at 37°C protected from light. The fluorometric absorbance was then measured at the Ex/Em of 400/505nm by using the microplate reader (Bio-Rad, Hercules, CA). The results were shown as relative fluorescence units (RFU) per microgram of protein.

### TUNEL assay

Myocardial apoptosis was detected using the terminal deoxynucleotide transferase dUTP nick end labeling (TUNEL) kit (Roche Life Science, Switzerland). The frozen sections were fixed in 4% paraformaldehyde and incubated with 0.2% TritonX-100 solution to break the cell membranes. The sections were then incubated with a mixture solution of enzyme solution and label solution for 1–1.5 hours shielded from light. The number of TUNEL-positive cells was counted under the fluorescence microscope.

### Statistical analysis

All values are shown as mean±SEM. When determining the statistical differences, unpaired student’s test was used between two groups, whereas ANOVA followed by Bonferroni multiple comparison test was applied among three or more groups. Kaplan-Meier curve was used to analyze the survival rates. P<0.05 was considered statistically significant. All the statistical analyses were performed using the GraphPad Prism (version 6.0).

## Supporting information

S1 Fig CatB activity was enhanced in hearts of CVB3-infected mice on day 7 postinfection. (A) The CatB activity was determined in the hearts of uninfected *Ctsb*^*-/-*^, *Cstc*^*-/-*^ and WT mice to verify the deletion and overexpression of CatB in *Ctsb*^*-/-*^ mice and *Cstc*^*-/-*^ mice. (n = 3 for each group; *P<0.05 *vs*. WT-con group; **P<0.01 *vs*. WT-con group; ###P<0.001 *vs*. *Ctsb*^*-/-*^-con group) (B) The enhanced cardiac CatB activity in WT+CVB3 group was significantly decreased in *Ctsb*^*-/-*^+CVB3 group but further increased in *Cstc*^*-/-*^+CVB3 group. (WT group: n = 3; WT+CVB3 group: n = 5; *Ctsb*^*-/-*^+CVB3 group: n = 3; *Cstc*^*-/-*^+CVB3 group: n = 3; *P<0.05 *vs*. WT group; #P<0.05 *vs*. WT+CVB3 group; &&&P<0.001 *vs*. *Ctsb*^*-/-*^+CVB3 group).
(TIF)

S2 Fig The cardiac structure, cardiac function and survival conditions have no difference among the uninfected *Ctsb*^*-/-*^, *Cstc*^*-/-*^ and WT mice. (A) Representative HE staining in hearts from the three kinds of mice. Scale bar: 50μm. (n = 3 for each group) (B-C) The echocardiographic data showed no statistical difference among the three groups. (WT group: n = 6; *Ctsb*^*-/-*^ group: n = 5; *Cstc*^*-/-*^ group: n = 5) (D) The Kaplan-Meier curve showed no death among the three groups in the indicated period. (WT group: n = 6; *Ctsb*^*-/-*^ group: n = 5; *Cstc*^*-/-*^ group: n = 5).
(TIF)

S3 Fig The effect of CatB on VMC was independent of altering cardiac viral replication. Genetic ablation of CatB or cystatin C had no impact on cardiac virus titers in mice 7 days (A) (WT+CVB3 group: n = 5; *Ctsb*^*-/-*^+CVB3 group: n = 5; *Cstc*^*-/-*^+CVB3 group: n = 5) and 28 days after CVB3 injection (B) (WT+CVB3 group: n = 4; *Ctsb*^*-/-*^+CVB3 group: n = 3; *Cstc*^*-/-*^+CVB3 group: n = 2).
(TIF)

S4 Fig The baseline inflammasome activation was similar in the hearts of the uninfected *Ctsb*^*-/-*^, *Cstc*^*-/-*^ and WT mice. (A) Western blot analysis of the expression of the components of the inflammasome including NLRP3, ASC, caspase-1 p-20 in hearts from the three groups of mice. (n = 3 for each group) (B) Cardiac caspase-1 activity was determined and presented as fold change compared with the WT mice. (n = 3 for each group) (C) Serum IL-1β level was detected by ELISA and had no difference among the three kinds of mice. (WT group: n = 5, *Ctsb*^*-/-*^ group: n = 5; *Cstc*^*-/-*^ group: n = 3).
(TIF)

S5 Fig The baseline percentage of cardiac TUNEL staining positive cells was similar in the hearts of the uninfected *Ctsb*^*-/-*^, *Cstc*^*-/-*^ and WT mice. (A) Representative cardiac TUNEL staining of mice with different genetic backgrounds. Scale bar: 100μm. (B) The statistical result of TUNEL exhibited no difference among the three kinds of mice. (n = 3 for each group).
(TIF)

S1 Table Echocardiographic Parameters of Mice with Indicated Treatment (Day 7 post-infection). IVS: interventricular septum; LVID: left ventricular internal dimension; LVPW: left ventricular posterior Wall; EF: ejection fraction; FS: fractional shortening; d: diastole; s: systole; CVB3: coxsackievirus B3; n = 10 for control; n = 10 for CVB3; Data presented as mean ± SE. *P<0.05 *vs*. control; **P<0.01 *vs*. control; ***P<0.001 *vs*. Control.
(DOC)

S2 Table Echocardiographic Parameters of Mice with Indicated Treatment (Day 28 post-infection). IVS: interventricular septum; LVID: left ventricular internal dimension; LVPW: left ventricular posterior Wall; EF: ejection fraction; FS: fractional shortening; d: diastole; s: systole; CVB3: coxsackievirus B3; n = 10 for control; n = 10 for CVB3; Data presented as mean ± SE. ^#^P<0.05 *vs*. Control; ^##^P<0.01 *vs*. Control; ^###^P<0.001 *vs*. Control.
(DOC)

S3 Table Echocardiographic Parameters of Mice with Indicated Treatment (Day 7, 14, 28 post-infection). IVS: interventricular septum; LVID: left ventricular internal dimension; LVPW: left ventricular posterior Wall; EF: ejection fraction; FS: fractional shortening; d: diastole; s: systole; WT: wild-type; CVB3: coxsackievirus B3; *Ctsb*^*-/-*^: cathepsin B knockout; *Cstc*^*-/-*^: cystatin C knockout. n = 10 for WT; n = 14 for WT+CVB3; n = 12 for *Ctsb*^*-/-*^+CVB3; n = 11 for *Cstc*^*-/-*^+CVB3; Data presented as mean ± SE. *P<0.05 *vs*. WT; **P<0.01 *vs*. WT; ***P<0.001 *vs*. WT; #P<0.05 *vs*. WT+CVB3; &&P<0.01 *vs*. *Ctsb*^*-/-*^+CVB3.
(DOC)
