# Supplementary figures and images for: Cathepsin B aggravates coxsackievirus B3-induced myocarditis through activating the inflammasome and promoting pyroptosis
Source: PLoS Pathog. 2018 Jan 23;14(1):e1006872. doi: 10.1371/journal.ppat.1006872 (PMC5809100; doi:10.1371/journal.ppat.1006872)

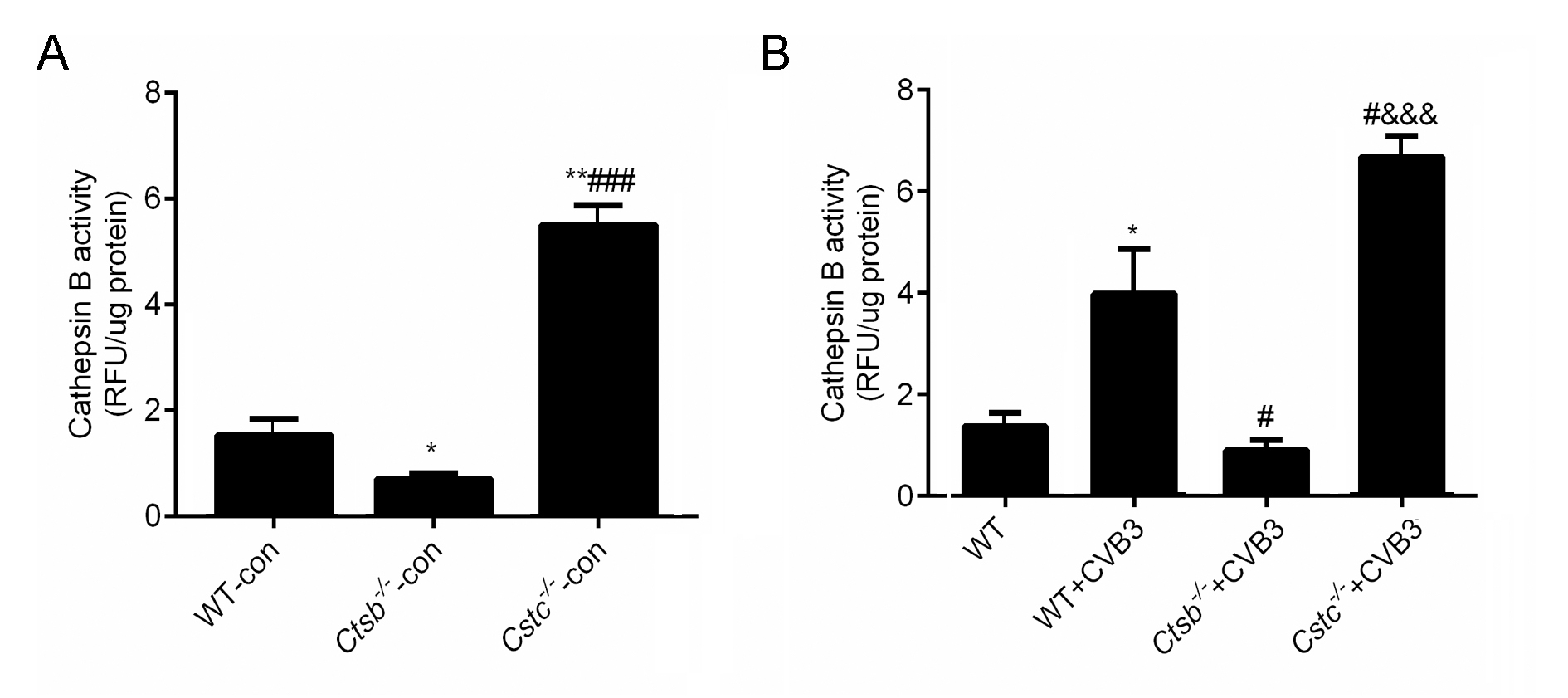

Supplement: S1 Fig — (A) The CatB activity was determined in the hearts of uninfected Ctsb-/-, Cstc-/- and WT mice to verify the deletion and overexpression of CatB in Ctsb-/- mice and Cstc-/- mice. (n = 3 for each group; *P<0.05 vs. WT-con group; **P<0.01 vs. WT-con group; ###P<0.001 vs. Ctsb-/--con group) (B) The enhanced cardiac CatB activity in WT+CVB3 group was significantly decreased in Ctsb-/-+CVB3 group but further increased in Cstc-/-+CVB3 group. (WT group: n = 3; WT+CVB3 group: n = 5; Ctsb-/-+CVB3 group: n = 3; Cstc-/-+CVB3 group: n = 3; *P<0.05 vs. WT group; #P<0.05 vs. WT+CVB3 group; &&&P<0.001 vs. Ctsb-/-+CVB3 group). (TIF) [file ppat.1006872.s001.tif]

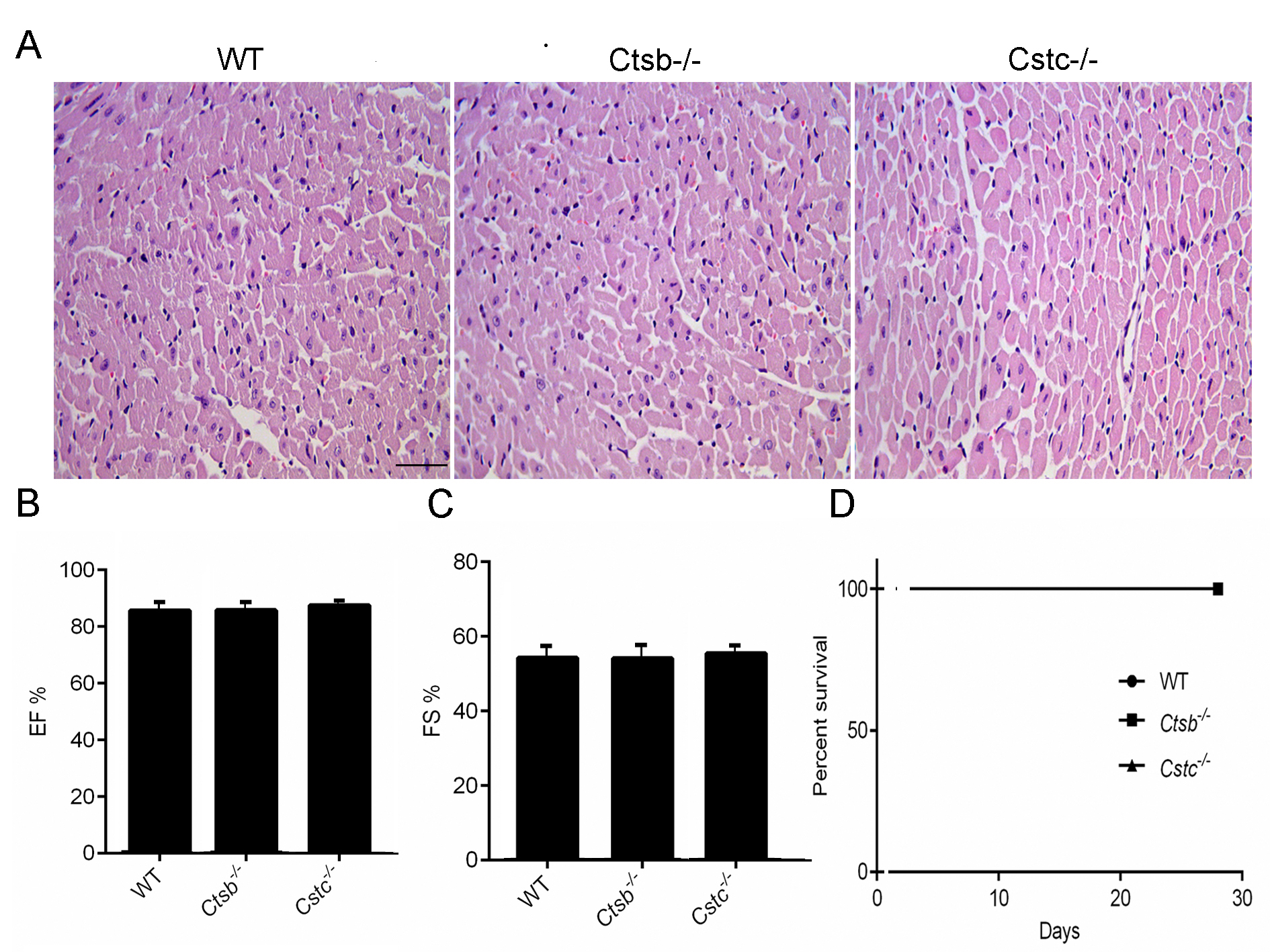

Supplement: S2 Fig — (A) Representative HE staining in hearts from the three kinds of mice. Scale bar: 50μm. (n = 3 for each group) (B-C) The echocardiographic data showed no statistical difference among the three groups. (WT group: n = 6; Ctsb-/- group: n = 5; Cstc-/- group: n = 5) (D) The Kaplan-Meier curve showed no death among the three groups in the indicated period. (WT group: n = 6; Ctsb-/- group: n = 5; Cstc-/- group: n = 5). (TIF) [file ppat.1006872.s002.tif]

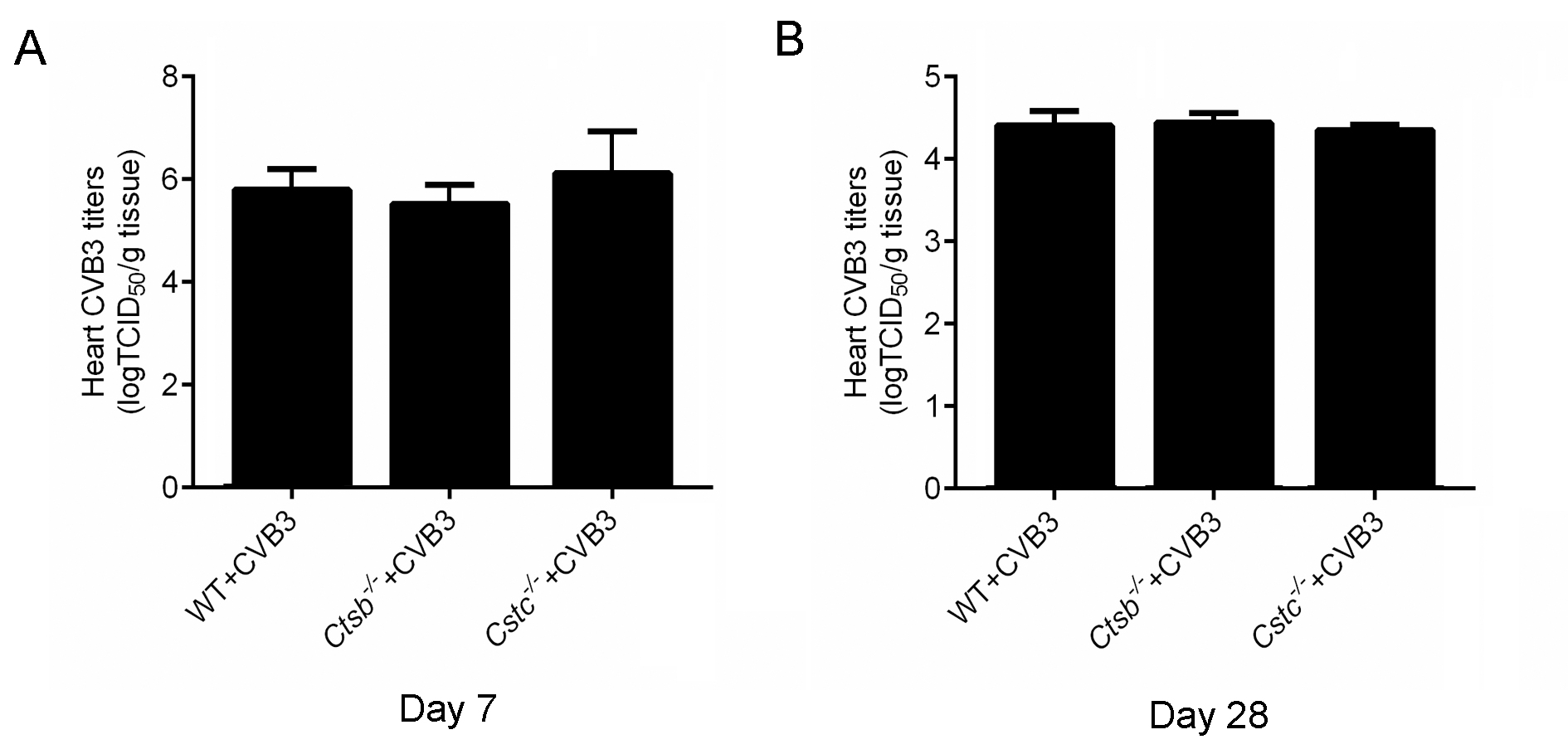

Supplement: S3 Fig — Genetic ablation of CatB or cystatin C had no impact on cardiac virus titers in mice 7 days (A) (WT+CVB3 group: n = 5; Ctsb-/-+CVB3 group: n = 5; Cstc-/-+CVB3 group: n = 5) and 28 days after CVB3 injection (B) (WT+CVB3 group: n = 4; Ctsb-/-+CVB3 group: n = 3; Cstc-/-+CVB3 group: n = 2). (TIF) [file ppat.1006872.s003.tif]

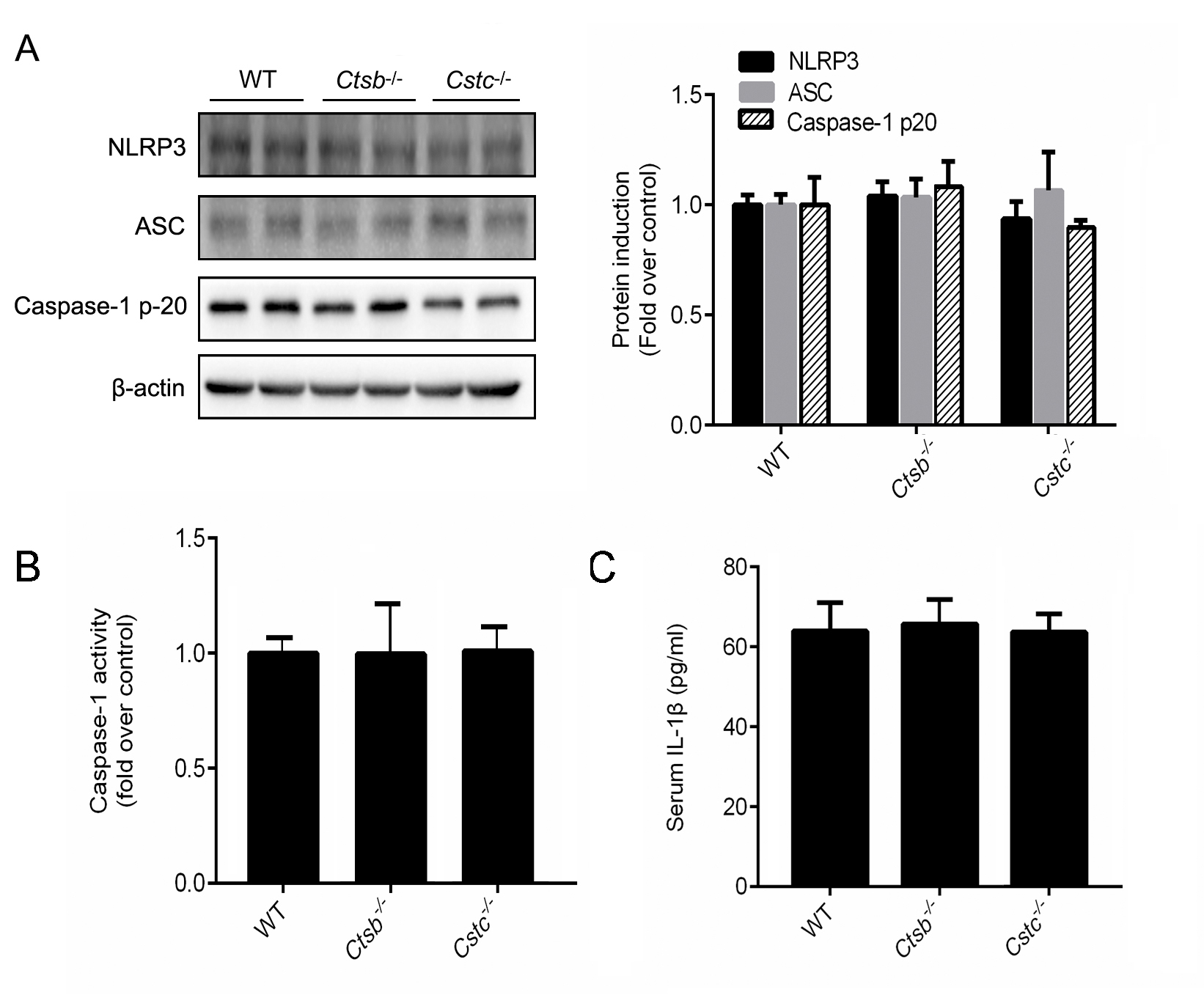

Supplement: S4 Fig — (A) Western blot analysis of the expression of the components of the inflammasome including NLRP3, ASC, caspase-1 p-20 in hearts from the three groups of mice. (n = 3 for each group) (B) Cardiac caspase-1 activity was determined and presented as fold change compared with the WT mice. (n = 3 for each group) (C) Serum IL-1β level was detected by ELISA and had no difference among the three kinds of mice. (WT group: n = 5, Ctsb-/- group: n = 5; Cstc-/- group: n = 3). (TIF) [file ppat.1006872.s004.tif]

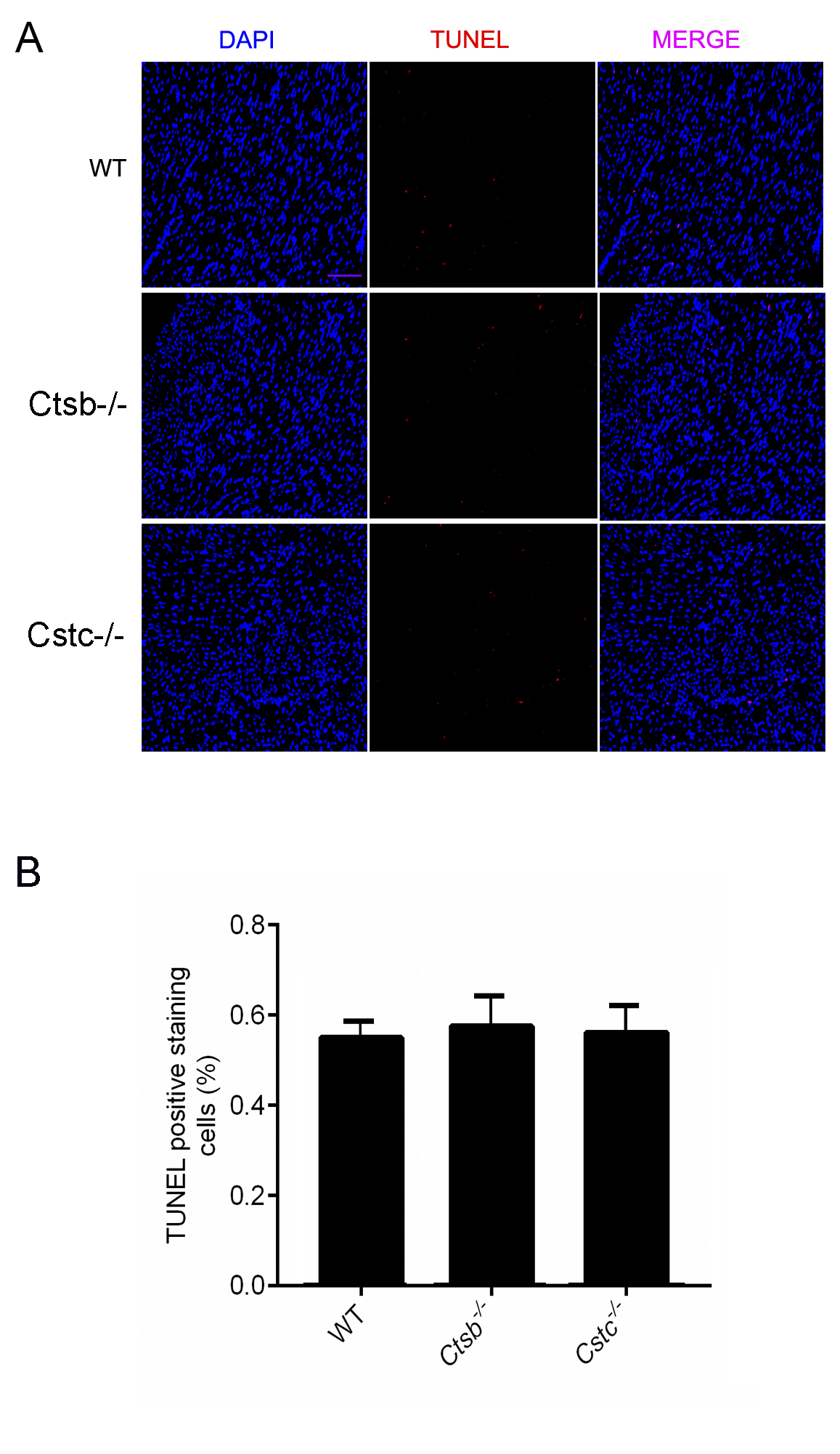

Supplement: S5 Fig — (A) Representative cardiac TUNEL staining of mice with different genetic backgrounds. Scale bar: 100μm. (B) The statistical result of TUNEL exhibited no difference among the three kinds of mice. (n = 3 for each group). (TIF) [file ppat.1006872.s005.tif]
