# Supplementary material for: Cathepsin B aggravates coxsackievirus B3-induced myocarditis through activating the inflammasome and promoting pyroptosis
Source: PLoS Pathog. 2018 Jan 23;14(1):e1006872. doi: 10.1371/journal.ppat.1006872 (PMC5809100; doi:10.1371/journal.ppat.1006872)
Supplement: S1 Table — IVS: interventricular septum; LVID: left ventricular internal dimension; LVPW: left ventricular posterior Wall; EF: ejection fraction; FS: fractional shortening; d: diastole; s: systole; CVB3: coxsackievirus B3; n = 10 for control; n = 10 for CVB3; Data presented as mean ± SE. *P<0.05 vs. control; **P<0.01 vs. control; ***P<0.001 vs. Control. (DOC) [file ppat.1006872.s006.doc]

**S1 Table.** Echocardiographic Parameters of Mice with Indicated Treatment (Day 7 post-infection).

|  | Control | CVB3 |
| --- | --- | --- |
| IVS-d (mm) | 1.05 ± 0.06 | 0.90 ± 0.28 |
| IVS-s (mm) | 1.71 ± 0.10 | 1.34 ± 0.39* |
| LVID-d (mm) | 2.60 ± 0.23 | 2.77 ± 0.40 |
| LVID-s (mm) | 0.96 ± 0.21 | 1.60 ± 0.32*** |
| LVPW-d (mm) | 1.07 ± 0.13 | 0.78 ± 0.21** |
| LVPW-s (mm) | 1.74 ± 0.20 | 1.12 ± 0.17*** |
| EF % | 92.13 ± 3.46 | 75.18 ± 5.26*** |
| FS % | 63.19 ± 6.34 | 42.52 ± 4.29*** |

IVS: interventricular septum; LVID: left ventricular internal dimension; LVPW: left ventricular posterior Wall; EF: ejection fraction; FS: fractional shortening; d: diastole; s: systole;

CVB3: coxsackievirus B3

n=10 for control; n=10 for CVB3

Data presented as mean ± SE. *P<0.05 *vs.* control; **P<0.01 *vs.* control; ***P<0.001 *vs.* Control
