# Supplementary material for: Cathepsin B aggravates coxsackievirus B3-induced myocarditis through activating the inflammasome and promoting pyroptosis
Source: PLoS Pathog. 2018 Jan 23;14(1):e1006872. doi: 10.1371/journal.ppat.1006872 (PMC5809100; doi:10.1371/journal.ppat.1006872)
Supplement: S2 Table — IVS: interventricular septum; LVID: left ventricular internal dimension; LVPW: left ventricular posterior Wall; EF: ejection fraction; FS: fractional shortening; d: diastole; s: systole; CVB3: coxsackievirus B3; n = 10 for control; n = 10 for CVB3; Data presented as mean ± SE. #P<0.05 vs. Control; ##P<0.01 vs. Control; ###P<0.001 vs. Control. (DOC) [file ppat.1006872.s007.doc]

**S2 Table.** Echocardiographic Parameters of Mice with Indicated Treatment (Day 28 post-infection).

|  | Control | CVB3 |
| --- | --- | --- |
| IVS-d (mm) | 1.00 ± 0.13 | 0.75 ± 0.20^##^ |
| IVS-s (mm) | 1.65 ± 0.13 | 1.15 ± 0.37^##^ |
| LVID-d (mm) | 2.73 ± 0.34 | 2.64 ± 0.41 |
| LVID-s (mm) | 1.15 ± 0.28 | 1.63 ± 0.45^#^ |
| LVPW-d (mm) | 1.14 ± 0.25 | 0.88 ± 0.28 |
| LVPW-s (mm) | 1.79 ± 0.24 | 1.27 ± 0.26^###^ |
| EF % | 88.69 ± 4.98 | 69.83 ± 13.52^##^ |
| FS % | 57.96 ± 6.93 | 38.79 ± 10.29^###^ |

IVS: interventricular septum; LVID: left ventricular internal dimension; LVPW: left ventricular posterior Wall; EF: ejection fraction; FS: fractional shortening; d: diastole; s: systole;

CVB3: coxsackievirus B3

n=10 for control; n=10 for CVB3

Data presented as mean ± SE. ^#^P<0.05 *vs.* Control; ^##^P<0.01 *vs.* Control; ^###^P<0.001 *vs.* Control
