# Supplementary material for: Cathepsin B aggravates coxsackievirus B3-induced myocarditis through activating the inflammasome and promoting pyroptosis
Source: PLoS Pathog. 2018 Jan 23;14(1):e1006872. doi: 10.1371/journal.ppat.1006872 (PMC5809100; doi:10.1371/journal.ppat.1006872)
Supplement: S3 Table — IVS: interventricular septum; LVID: left ventricular internal dimension; LVPW: left ventricular posterior Wall; EF: ejection fraction; FS: fractional shortening; d: diastole; s: systole; WT: wild-type; CVB3: coxsackievirus B3; Ctsb-/-: cathepsin B knockout; Cstc-/-: cystatin C knockout. n = 10 for WT; n = 14 for WT+CVB3; n = 12 for Ctsb-/-+CVB3; n = 11 for Cstc-/-+CVB3; Data presented as mean ± SE. *P<0.05 vs. WT; **P<0.01 vs. WT; ***P<0.001 vs. WT; #P<0.05 vs. WT+CVB3; &&P<0.01 vs. Ctsb-/-+CVB3. (DOC) [file ppat.1006872.s008.doc]

**S3 Table.** Echocardiographic Parameters of Mice with Indicated Treatment (Day 7, 14, 28 post-infection).

|  | WT | WT+  CVB3 | *Ctsb^-/-^*+CVB3 | *Cstc^-/-^*+CVB3 | WT | WT+  CVB3 | *Ctsb^-/-^*+CVB3 | *Cstc^-/-^*+CVB3 | WT | WT+  CVB3 | *Ctsb^-/-^*+CVB3 | *Cstc^-/-^*+CVB3 |
| --- | --- | --- | --- | --- | --- | --- | --- | --- | --- | --- | --- | --- |
|  | Dat 7 | | | | Day 14 | | | Day 28 | | | | |
| IVS-d (mm) | 0.90±0.16 | 0.71±0.19* | 0.76±0.18 | 0.93±0.31# | 0.91±0.08 | 0.85±0.22 | 0.72±0.13 | 0.93±0.30 | 1.04±0.17 | 0.79±0.23* | 0.74±0.22 | 0.77±0.07 |
| IVS-s (mm) | 1.53±0.19 | 1.02±0.28*** | 1.16±0.24 | 1.39±0.42 | 1.52±0.11 | 1.20±0.14*** | 1.16±0.18 | 1.29±0.35 | 1.65±0.13 | 1.23±0.41** | 1.16±0.27 | 1.06±0.21 |
| LVID-d (mm) | 2.49±0.22 | 2.80±0.37* | 2.96±0.62 | 2.65±0.55 | 2.64±0.40 | 2.47±0.45 | 2.69±0.55 | 2.47±0.68 | 2.55±0.40 | 2.60±0.44 | 3.00±0.59 | 3.69±0.47# |
| LVID-s (mm) | 0.94±0.18 | 1.81±0.30*** | 1.84±0.70 | 1.78±0.48 | 1.12±0.38 | 1.64±0.32** | 1.59±0.57 | 1.81±0.53 | 1.12±0.28 | 1.54±0.50* | 1.83±0.72 | 2.50±0.50# |
| LVPW-d (mm) | 1.05±0.27 | 0.74±0.39* | 0.72±0.21 | 0.87±0.27 | 1.08±0.28 | 0.85±0.37 | 0.71±0.16 | 1.10±0.47 | 1.17±0.26 | 0.93±0.31 | 0.78±0.15 | 0.99±0.01 |
| LVPW-s (mm) | 1.70±0.22 | 1.07±0.33*** | 1.12±0.32 | 1.11±0.21 | 1.72±0.27 | 1.15±0.39** | 1.14±0.29 | 1.18±0.24 | 1.79±0.23 | 1.33±0.25*** | 1.21±0.25 | 1.24±0.03 |
| EF % | 92.08±2.63 | 66.73±6.78*** | 69.86±14.89 | 62.79±11.00 | 88.67±5.69 | 63.89±9.22*** | 73.98±11.86# | 53.86±14.65&& | 87.76±4.48 | 72.97±14.22* | 70.94±15.77 | 61.63±7.50 |
| FS % | 62.62±4.69 | 35.69±5.10*** | 39.79±12.70 | 32.97±7.86 | 58.56±9.46 | 33.43±6.65*** | 42.21±9.38# | 26.92±8.52&& | 56.22±5.97 | 41.49±10.69** | 40.89±13.29 | 32.56±4.98 |

IVS: interventricular septum; LVID: left ventricular internal dimension; LVPW: left ventricular posterior Wall; EF: ejection fraction; FS: fractional shortening; d: diastole; s: systole

WT: wild-type; CVB3: coxsackievirus B3; *Ctsb^-/-^*: cathepsin B knockout; *Cstc^-/-^*: cystatin C knockout.

n=10 for WT; n=14 for WT+CVB3; n=12 for *Ctsb^-/-^*+CVB3; n=11 for *Cstc^-/-^*+CVB3

Data presented as mean ± SE. *P<0.05 *vs.* WT; **P<0.01 *vs.* WT; ***P<0.001 *vs.* WT; #P<0.05 *vs.* WT+CVB3; &&P<0.01 *vs.* *Ctsb^-/-^*+CVB3
